# Ethyl Gallate Dual-Targeting PTPN6 and PPARγ Shows Anti-Diabetic and Anti-Obese Effects

**DOI:** 10.3390/ijms23095020

**Published:** 2022-04-30

**Authors:** Dohee Ahn, Jinsoo Kim, Gibeom Nam, Xiaodi Zhao, Jihee Kwon, Ji Young Hwang, Jae Kwan Kim, Sun-Young Yoon, Sang J. Chung

**Affiliations:** 1 School of Pharmacy, Sungkyunkwan University, Suwon 16419, Korea; ehgml94@naver.com (D.A.); neto543@naver.com (J.K.); skarlqja12@g.skku.edu (G.N.); zhaoxiaodi1019@gmail.com (X.Z.); smailemaster@naver.com (J.Y.H.); jk941002@naver.com (J.K.K.); 2 Department of Biopharmaceutical Convergence, Sungkyunkwan University, Suwon 16419, Korea; yg1549@naver.com; 3 Department of Cosmetic Science, Kwangju Women’s University, Gwangju 62396, Korea; dalae1104@gmail.com

**Keywords:** adipogenesis, 3T3-L1 adipocyte, ethyl gallate, natural product, PTPN6, AMPK, PPARγ antagonist

## Abstract

The emergence of the high correlation between type 2 diabetes and obesity with complicated conditions has led to the coinage of the term “diabesity”. AMP-activated protein kinase (AMPK) activators and peroxisome proliferator-activated receptor (PPARγ) antagonists have shown therapeutic activity for diabesity, respectively. Hence, the discovery of compounds that activate AMPK as well as antagonize PPARγ may lead to the discovery of novel therapeutic agents for diabesity. In this study, the knockdown of PTPN6 activated AMPK and suppressed adipogenesis in 3T3-L1 cells. By screening a library of 1033 natural products against PTPN6, we found ethyl gallate to be the most selective inhibitor of PTPN6 (*K*_i_ = 3.4 μM). Subsequent assay identified ethyl gallate as the best PPARγ antagonist (IC_50_ = 5.4 μM) among the hit compounds inhibiting PTPN6. Ethyl gallate upregulated glucose uptake and downregulated adipogenesis in 3T3-L1 cells as anticipated. These results strongly suggest that ethyl gallate, which targets both PTPN6 and PPARγ, is a potent therapeutic candidate to combat diabesity.

## 1. Introduction

The twin upsurge in diabetes and obesity is a severe global crisis [1]. Obesity and overweight reportedly increase the risk of progression to type 2 diabetes mellitus (T2DM) by three and seven-fold, respectively [2]. Moreover, approximately 85.2% of people suffering from T2DM also show symptoms of obesity and are overweight [3]. The term “diabesity” represents a close relationship between diabetes and obesity [4]. Hence, it is essential to discover potent therapeutic agents effective against both diabetes and obesity.

Various treatments exist for patients suffering from diabesity. Bariatric surgery might be the optimum treatment option for patients with diabesity; however, many patients do not prefer the surgical procedure because it is invasive, impermissible, expensive, or can lead to various complications. Hence, these patients commonly receive pharmacotherapies. As inappropriate control of T2DM can increase the risk of macrovascular, microvascular, and metabolic complications; proper glycemic control is essential for patients with diabesity [5]. Several anti-diabetic medications, including insulin, can increase body weight and worsen diabesity [6]. A few drugs, such as glucagon-like peptides-1 (GLP-1) receptor agonists and sodium-glucose transporter-2 (SGLT-2) inhibitors, have been proposed for the treatment of diabesity. However, due to the several adverse effects of these drugs, the discovery of novel therapeutic agents with minimal side effects is an area of major interest [7].

Peroxisome proliferator-activated receptor (PPARγ) agonists, such as thiazolidinediones (TZDs), used in the treatment of diabetes, have also been reported to show unwanted side effects, including weight gain, cardiovascular disease, fluid retention, bone fracture, and bladder cancer. As a result, the investigation of PPARγ antagonists has been suggested as a key strategy to combat diabesity [8,9,10]. PPARγ antagonists bind to the ligand-binding domain (LBD) with an allosteric switch in the activation helix and promote co-repressor binding [11]. Therefore, PPARγ antagonists can reduce the side effects of PPARγ agonism while maintaining the blockage of cyclin-dependent kinase 5 (CDk5)-mediated phosphorylation for anti-diabetic effects in adipocytes [12,13]. Since both betulinic acid and Gleevec antagonizing PPARγ were effective in both diabetes and obesity, exploring novel PPARγ antagonists could be a promising approach for the treatment of diabesity [8,10].

AMP-activated protein kinase (AMPK) has also been observed as a potential target for the treatment of diabesity [14,15]. Typically, AMPK stimulates glucose uptake into the cells by enhancing the translocation of glucose transporters (GLUT) such as GLUT1 and GLUT4 [14]. Moreover, AMPK is involved in adipocyte differentiation as an upstream regulator of PPARγ, indicating that AMPK activators can exert an anti-obesity effect [15,16,17]. Various pharmacological agents reported as AMPK activators are reportedly effective against diabetes or obesity [15,18]. Metformin, widely used as an anti-diabetic drug, also reportedly activates AMPK [15]. Although metformin has not yet been approved for anti-obesity treatment, recent studies have demonstrated its effectiveness in reducing weight [19].

Protein tyrosine phosphatases (PTPs) have recently emerged as putative therapeutic targets for several metabolic diseases [20]. In our previous studies, we showed that several PTPs, including DUSP9, PTPN9, and PTPN11, may be targeted to treat T2DM during their downregulation by increasing the phosphorylation of AMPK [21,22]. Among PTPs, protein tyrosine phosphatase non-receptor type 6 (PTPN6) has also been a target of major interest for metabolic diseases [23,24]. Deletion of hepatocytic PTPN6 protects mice from hepatic insulin resistance caused by a high-fat diet (HFD) and severe liver inflammation in steatosis; however, how the role of PTPN6 in adipocytes is associated with metabolic functions remains unclear [25]. In this study, we identified PTPN6 as a new AMPK-related target for diabesity in 3T3-L1 cells by using siRNA.

Collectively, compounds that inhibit PTPN6 and antagonize PPARγ are believed to exhibit potential effects on T2DM and obesity. By implementing a 6,8-difluoro-4-methylumbelliferyl phosphate (DiFMUP)-based screening system and luciferase reporter gene assay using an in-house library of 1033 natural products, we identified ethyl gallate (EG), targeting both PTPN6 and PPARγ. EG is found in several medicinal plants and natural food products such as wines, fruits, and nuts [26,27,28,29]. EG is known for its antioxidant, anticancer, anti-inflammatory, and protective effects against atherosclerosis [30,31,32,33]. However, its effects on diabetes and obesity have not been studied. We found that EG, targeting both PTPN6 and PPARγ, stimulated glucose uptake by activating AMPK and inhibited adipogenesis in 3T3-L1 cells. These results suggest that EG is a potent therapeutic agent for diabesity.

## 2. Results

### 2.1. The Depletion of PTPN6 by siRNA Knockdown Increases AMPK Phosphorylation

**Figure 1:**
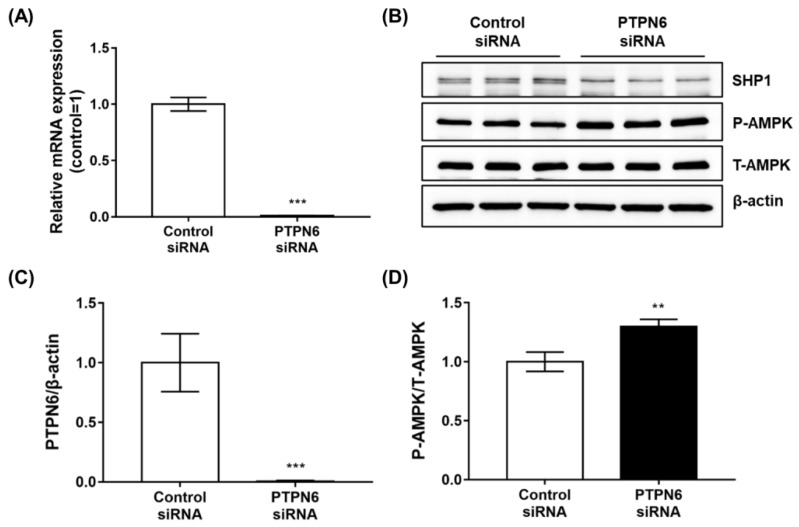
The depletion of PTPN6 by siRNA knockdown increases AMPK phosphorylation. 3T3-L1 preadipocytes were transfected with PTPN6 siRNA for 48 h. After cell lysis, the expression of PTPN6 was analyzed by quantitative real-time-polymerase chain reaction (qRT-PCR) (**A**) and Western blotting (**B**,**C**). Changes in the phosphorylation of AMPK following the knockdown of PTPN6 (also named SHP1) were analyzed by Western blotting (**B**). Quantification was performed and normalized to control using CSAnalyzer 4 (**C**,**D**). Data are shown as mean ± standard deviation (*n* = 3). ** *p* < 0.01, *** *p* < 0.001. Statistical significance was analyzed by a two-tailed unpaired *t*-test.

Several protein phosphatases, such as PTPN9, DUSP9, and PTPN11, have been identified as AMPK-related targets for type 2 diabetes [21,22]. To extend the results of these studies in the discovery of new targets with novel inhibitors, PTPN6 was evaluated as a potential target for anti-diabetic effects. To investigate the role of PTPN6 in 3T3-L1 fat cells, we performed a siRNA knockdown to downregulate PTPN6 expression. After siRNA treatment for 48 h in 3T3-L1 preadipocytes, mRNA expression of PTPN6 was measured by quantitative real-time-polymerase chain reaction (qRT-PCR). PTPN6 mRNA expression decreased by more than 95% compared to that in the negative control (Figure 1A). Protein expression of PTPN6, shown as the lowest of the two bands performed by Western blotting, also decreased by more than 95%, corresponding to qRT-PCR levels (Figure 1B,C). As PTPN6 expression decreased, the phosphorylation of AMPK, which stimulates GLUT4 translocation in 3T3-L1 adipocytes, increased significantly (Figure 1D). These results indicated that PTPN6 in fat cells could serve as a therapeutic target for type 2 diabetes via the AMPK signaling pathway.

### 2.2. The Knockdown of PTPN6 by siRNA Suppresses Adipogenesis of 3T3-L1 Cells

**Figure 2:**
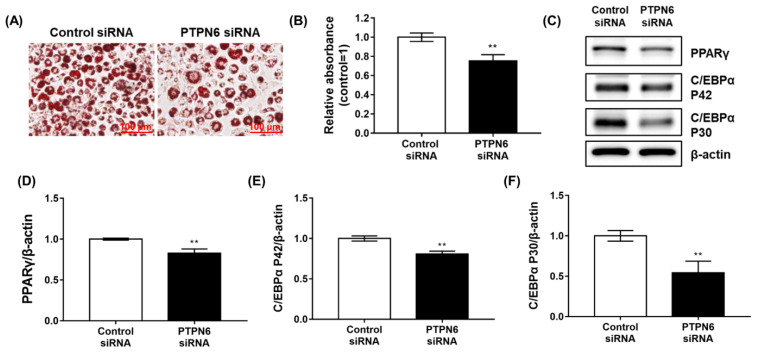
siRNA knockdown of PTPN6 suppresses adipogenesis of 3T3-L1 preadipocytes. After the knockdown of PTPN6 using siRNA for 48 h, 3T3-L1 preadipocytes were differentiated by DMI-induction for 6 days (DMI: dexamethasone, methylisobutylxanthine, and insulin). Lipid droplets of 3T3-L1 adipocytes were stained using the Oil red O working solution and visualized. Scale bar, 100 μm. (**A**). Quantitative data on the lipid content were analyzed with extracted Oil red O using 100% isopropanol (**B**). For Western blot analysis, the cells were lysed after 48 h of differentiation. Western blot was performed using anti-PPARγ, C/EBPα, and β-actin antibodies (**C**). Quantification was performed for PPARγ (**D**), C/EBPα P42 (**E**), and C/EBPα P30 (**F**), and each was normalized to control using CSAnalyzer 4. Data are shown as mean ± standard deviation (*n* = 3). ** *p* < 0.01. Statistical significance was analyzed by a two-tailed unpaired *t*-test.

The activation of AMPK, a suggested target for diabesity, is known to suppress the adipogenesis of 3T3-L1 cells by downregulating PPARγ and CCAAT/enhancer-binding protein α (C/EBPα) [16,17]. Since AMPK is activated after the knockdown of PTPN6, PTPN6 was also evaluated as a target for obesity using siRNA. After the treatment with PTPN6 siRNA for 48 h, 3T3-L1 preadipocytes were differentiated by DMI-induction (DMI: dexamethasone, methylisobutylxanthine, and insulin) for 6 days. Lipid droplets of 3T3-L1 cells were stained using the Oil red O working solution and fixed with 4% paraformaldehyde (Figure 2A). Quantitative data showed that the knockdown of PTPN6 weakly but significantly inhibited adipogenesis of 3T3-L1 cells (approximately 25% inhibition), as shown in Figure 2A (Figure 2B). To explore the effects of PTPN6 knockdown at the early stage of differentiation, changes in the level of the early adipogenic factors, such as PPARγ and C/EBPα, were analyzed by Western blotting on day 2 of differentiation (Figure 2C). PPARγ and C/EBPα were decreased slightly, but significantly by approximately 17% for PPARγ (Figure 2D), 19% for C/EBPα P42 (Figure 2E), and 46% for C/EBPα P30 (Figure 2F) compared to the negative control. These results suggested that PTPN6 might be involved in regulating adipogenesis of 3T3-L1 preadipocytes.

### 2.3. Selection and Validation of Ethyl Gallate as a PTPN6 Inhibitor

**Figure 3:**
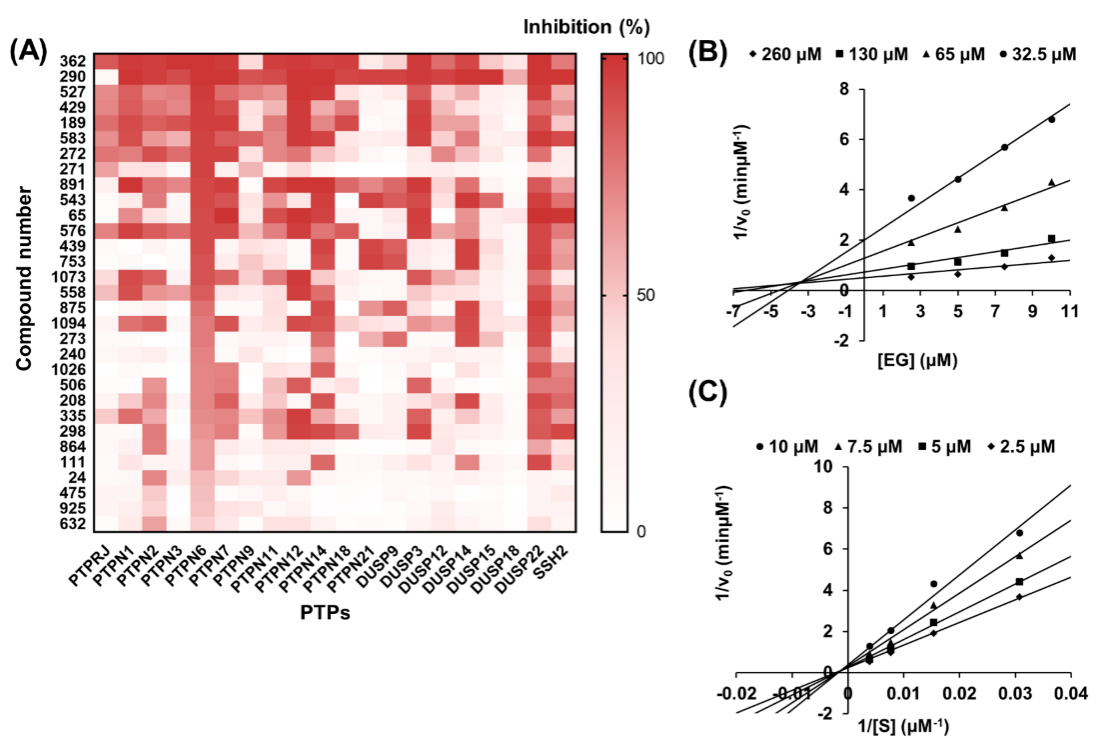
Selection and validation of ethyl gallate (EG) as a PTPN6 inhibitor. For the selection of PTPN6 inhibitors, a 1033 natural products library (20 μM for each compound) was screened against 20 PTPs. A substrate, 6,8-difluoro-4-methylumbelliferyl phosphate (DiFMUP), which is hydrolyzed to DiFMU after reactions with protein tyrosine phosphatases (PTPs), was used at the concentration of 2 × *K*_m_ for measuring enzymatic activities. The increase in fluorescence intensity at Ex/Em = 355/460 nm was monitored by a microplate reader using a 96-well black plate. Results of screening with over 60% inhibition for PTPN6 are reported as heatmap analysis using GraphPad Prism. The compound names corresponding to the compound numbers are listed in Table S1. (**A**). To determine the inhibition constant (*K*_i_) and type of inhibition of EG (271), various concentrations of EG (2.5, 5, 7.5, and 10 μM) were added to the pH 7.0 reaction buffer containing different concentrations (0.5 × *K*_m_, 1 × *K*_m_, 2 × *K*_m_, and 4 × *K*_m_; *K*_m_ = 86.26 μM) of DiFMUP followed by treatment of 2.0 nM PTPN6. The *K*_i_ and type of inhibition were derived from a Dixon plot (**B**) and a Lineweaver-Burk plot (**C**) for EG inhibition.

In an approach to discover new compounds with anti-diabetic or anti-obesity effects, natural products inhibiting PTPN6 were searched from an in-house library. Full-length human PTPN6 was expressed with an N-terminal hexahistidine (His6) tag using the *Escherichia coli* expression system and purified using TALON^®^ affinity resin (Figures S1 and S2). The enzymatic activity of purified PTPN6 was evaluated by Michaelis–Menten plot and Lineweaver-Burk plot. Kinetic parameters expressed as *K*_m_, *V*_max_, *k*_cat_, and *k*_cat_/*K*_m_ values *k*_cat_/*K*_m_ were 86.26 μM, 4.07 μM/min, 2.03 × 10^3^ min^−1^, and 2.32 × 10^2^ μM^−1^ min^−1^, respectively (Figure S3). Then, a library consisting of 1033 natural products was screened against PTPN6 under the optimized condition. The same screening was performed against an additional 19 PTPs to assess the inhibition selectivity of the natural products. Combining these results, inhibitors of PTPN6 showing activity of over 60% were listed in decreasing order of percent inhibition, as shown in Figure 3A. Out of the 31 hit compounds, EG (271) presented the highest selectivity against PTPN6 at a 20 μM concentration. EG showed an estimated half-inhibitory concentration (IC_50_) at 9.0 μM from the sigmoid plot and positive cooperativity with a Hill coefficient (n_H_) of 1.7 (Figure S4). Further, kinetic analysis using Dixon and Lineweaver-Burk plots revealed that EG appeared to be a mixed inhibitor with an inhibition constant (*K*_i_) of 3.4 μM (Figure 3B,C).

### 2.4. PPARγ Antagonistic Effect of Ethyl Gallate

**Figure 4:**
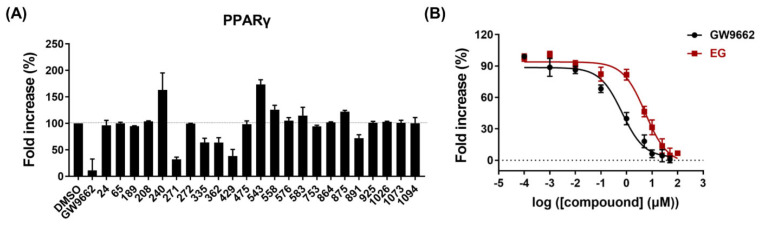
Identification of PPARγ antagonistic effect of ethyl gallate (EG). After a 6 h co-transfection with pCMV6-hPPARG-GFP and pPPRE-TK-luc, CHO cells were treated with compounds in the presence of 2 μM rosiglitazone and incubated for 24 h. (**A**) PPARγ antagonistic activities against hit compounds, exhibiting PTPN6 inhibition. Cells were treated with each compound at 20 μM concentration. The compound names corresponding to the compound numbers are listed in Table S1. Data are shown as mean ± standard deviation (*n* = 2). (**B**) Concentration-dependent PPARγ antagonistic activities of EG. Cells were treated with EG or GWW9662 at various concentrations (0.0001, 0.001, 0.01, 0.1, 1, 5, 10, 25, 50, and 100 μM). The IC_50_ values represent the concentrations of compounds that inhibited 50% of the response induced by 2 μM rosiglitazone. Data are shown as mean ± standard deviation (*n* = 3).

To determine the PPARγ antagonistic activities of PTPN6 inhibitors, a PPARγ transactivation assay was performed based on a luciferase reporter gene assay. CHO cells were co-transfected with pCMV6-hPPARG-GFP and pPPRE-TK-luc for 6 h, and then cells were treated with each compound at a concentration of 20 μM in the presence of 2 μM rosiglitazone for 24 h. Among the 31 hit compounds inhibiting PTPN6, eight compounds showed high cytotoxicity (cell viability < 70%) (Figure S5). Of the remaining 23 compounds, five compounds (EG (271), icariside I (335), juglone (362), myricetrin (429), and agaric acid (891)) antagonized PPARγ in the 28–68% range, where EG showed the highest PPARγ antagonistic effect (68%) (Figure 4A). GW9662, used as a positive control, exhibited an 89% antagonistic effect at 20 μM concentration. Treatment with various concentrations of EG and GW9662 suppressed the agonism of rosiglitazone in a concentration-dependent manner, with IC_50_ values of 5.4 μM and 0.7 μM, respectively (Figure 4B). These results indicated that EG is a potent antagonist of PPARγ.

### 2.5. Docking Model of EG on PTPN6 and PPARγ

Next, an in silico docking study was conducted to investigate the molecular basis of EG and other screened compounds against PTPN6 and PPARγ. Catalytic sites of PTPs are highly conserved and share signature motifs. “P-loop” consists of the HCX5R motif, where its nucleophilic cysteine is essential for the coordination and catalytic dephosphorylation of phosphorylated substrates [34,35]. Another conserved motif is the WPD loop. When the substrate binds, the flexible WPD loop encloses the P-loop and participates in the dephosphorylation of the catalytic Cys through a general acid–base catalysis mechanism by aspartate residue [36,37]. Multiple X-ray crystal structures of PTPN6 have been reported [38,39]. PTPN6 and PTPN11 (SHP2) possess two unique Src homology 2 domains (N-SH2 and C-SH2), thus regulating dephosphorylation activity. Both activated and inactivated PTPN6 were used for docking analysis.

**Figure 5:**
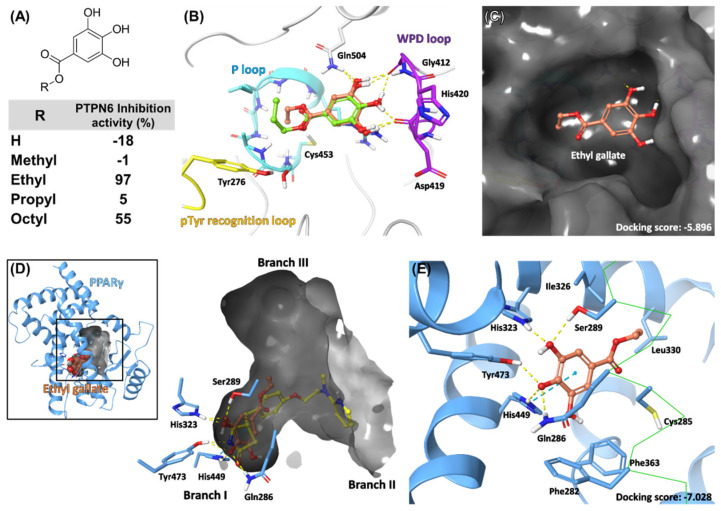
Docking model of ethyl gallate (EG) on PTPN6 and PPARγ. (**A**) Structure of gallate derivatives and screening results of gallate derivatives on PTPN6. (**B**) Docking model of EG (orange) and propyl gallate (pale green) on PTPN6 (white). Ligands are presented as ball-and-stick models. Important motifs and residues of PTPN6 are highlighted in color. Hydrogen bonds are represented as yellow dashed lines. (**C**) Surface representation of PTPN6 in gray. (**D**) Docking model of EG (orange) and the X-ray conformation of rosiglitazone (yellow) on PPARγ (sky blue). The orthosteric pocket is represented as a gray surface. (**E**) Multiple H-bonds between EG and residues in branch I. π–π stacking interaction is represented as blue dashed lines. For visual inspection, the representation of helix3 is simplified as a green CA trace line.

In the docking model of EG with PTPN6, the 1,2,3-trihydroxybenzene group formed H-bonds with the backbone carbonyl groups of the WPD loop (Asp419 and Gly421) and Gln504. Conserved arginine on the P-loop (Arg459) is located under the phenyl ring and could contribute via cation–π interactions. The ester group is headed toward the P-loop, and ethyl carbons tightly fit into the central groove (Figure 5B,C). Interestingly, EG specifically inhibited PTPN6 compared to the other four gallate derivatives in PTP screening, indicating that the length of the carbon chain is crucial for interaction with PTPN6 (Figure 5A). The propyl group in the propyl gallate could not fit into the catalytic groove in our docking model, suggesting a clear correlation with the inhibition activities (Figure 5B).

As described above, most PPARγ ligands bind to LBD. This orthosteric pocket is one of the largest in the nuclear receptor superfamily (~1400 Å^3^) and branches into a "T" or "Y" shape [40,41]. The primary binding mechanism of synthetic ligands, including TZD [40], partial agonists [42], and covalent antagonists [41], involves multiple electrostatic interactions with residues in branch I, where important activation function-2 (AF-2) and helix12 are located (Figure 5D). The docking model of EG in PPARγ showed that the 1,2,3-trihydroxybenzene group formed H-bonds with Gln286, Ser289, His323, His449, and Tyr473, which are identical to the TZD moiety of rosiglitazone [40]. π–π stacking with His449 and hydrophobic interactions of the ethyl tail grant additional interaction between EG and PPARγ (Figure 5E).

### 2.6. EG Treatment Upregulates Glucose Uptake and Activates AMPK in 3T3-L1 Adipocytes

**Figure 6:**
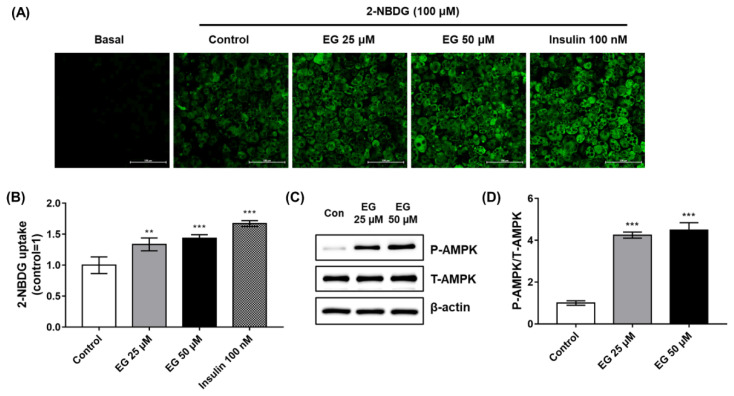
Ethyl gallate (EG) treatment upregulates glucose uptake and activates AMPK in 3T3-L1 adipocytes. Preadipocytes of 3T3-L1 cells were differentiated by DMI-induction (DMI: dexamethasone, methylisobutylxanthine, and insulin) for 6 days. Fully differentiated 3T3-L1 cells were incubated overnight with low-glucose Dulbecco’s modified Eagle’s medium (DMEM) for starvation, followed by treatment of EG (6 h) or insulin (30 min) in glucose-depleted DMEM with 0.1% DMSO. Subsequently, they were treated using 100 μM 2-NBDG for 1 h. Fluorescence intensity was detected at Ex/Em = 485/535 nm by confocal microscopy (**A**) or microplate reader (**B**) after washing with cold DPBS. Scale bar, 100 μm. For Western blot analysis, cells were lysed and analyzed after treatment of EG under the same condition as the 2-NBDG uptake assay. Western blotting was performed using anti-P-AMPK, T-AMPK, and β-actin antibodies (**C**). Quantification was performed for P-AMPK, T-AMPK, and normalized to control using CSAnalyzer 4 (**D**). Data are shown as mean ± standard deviation (*n* = 3 or 4). ** *p* < 0.01. *** *p* < 0.001. Statistical significance was analyzed by one-way ANOVA for multiple comparisons followed by Tukey–Kramer statistical test.

To evaluate the anti-diabetic effects of EG, a glucose uptake assay, which is based on the detection of 2-[N-(7-nitrobenz-2-oxa-1,3-diazol-4-yl)amino]-2-deoxyglucose (2-NBDG), a fluorescent glucose analog, was performed in 3T3-L1 cells. After treatment with 2-NBDG, the brightness of images captured by confocal microscopy increased in the insulin or EG treatment groups in a concentration-dependent manner compared to that in the control, 0.1% dimethyl sulfoxide (DMSO) treatment groups (Figure 6A). To quantify the fluorescence intensity of 2-NBDG in the cells, 2-NBDG uptake assays were performed in black 96-well plates and detected using a microplate reader. The results showed the same tendency as in Figure 4A, in which insulin and EG significantly enhanced the 2-NBDG uptake ability of differentiated 3T3-L1 cells (Figure 6B). To further verify the mechanism of EG in stimulating glucose uptake, 3T3-L1 cells were treated with EG under the same conditions as those in 2-NBDG uptake assays, and the expression levels of total and phosphorylated AMPK were detected using Western blot analysis. Consistent with the results of the 2-NBDG uptake assay, phosphorylated AMPK normalized to total AMPK significantly increased in a concentration-dependent manner (Figure 6C,D). These results suggested that an increase in AMPK phosphorylation by EG targeting PTPN6 might contribute to the improvement of glucose homeostasis in type 2 diabetes.

### 2.7. EG Treatment Suppresses the Early Stage of Adipogenesis in 3T3-L1 Cells

**Figure 7:**
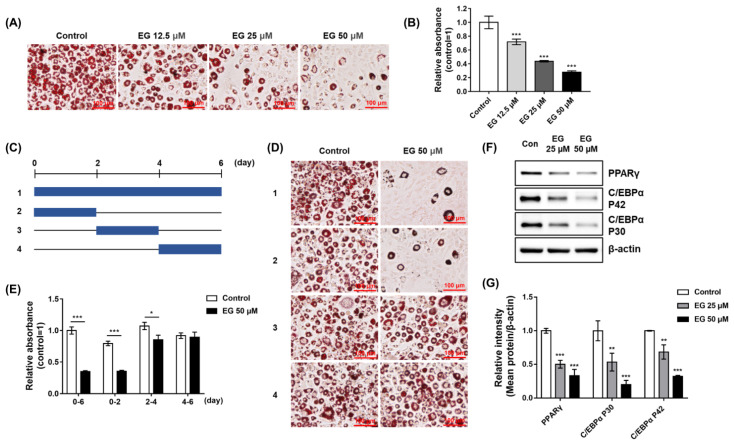
Anti-adipogenic effect of ethyl gallate (EG) at the early stage of differentiation. Preadipocytes of 3T3-L1 cells were differentiated by DMI-induction (DMI: dexamethasone, methylisobutylxanthine, and insulin) and treated with various concentrations of EG (12.5 μM, 25 μM, and 50 μM) simultaneously. To analyze which stages are affected by the treatment with EG, 3T3-L1 cells were treated with 50 μM EG for 0–6 days (1), 0–2 days (2), 2–4 days (3), or 4–6 days (4) during DMI-induced differentiation (**C**). On day 6 of differentiation, lipid droplets of 3T3-L1 adipocytes were stained using the Oil red O working solution and visualized. Scale bar, 100 μm (**A**,**D**). Quantitative data on the lipid content were analyzed using extracted Oil red O with 100% isopropanol (**B**,**E**). For analysis of early adipogenic factors, cells were lysed and analyzed by Western blot using anti-PPARγ, C/EBPα, and β-actin antibodies after treating with EG for 48 h during DMI-induced differentiation (**F**). Quantification was performed, and each was normalized to control using CSAnalyzer 4 (**G**). Data are shown as mean ± standard deviation (*n* = 3). * *p* < 0.05, ** *p* < 0.01, *** *p* < 0.001. Statistical significance was analyzed by one-way ANOVA for multiple comparisons followed by Tukey–Kramer statistical test.

To elucidate the anti-adipogenic effects of EG in 3T3-L1 cells, Oil red O staining was performed after differentiation with EG treatment. As shown in Figure 7A, lipid droplet accumulation decreased in the EG treatment groups in a concentration-dependent manner. For quantitative analysis, the Oil red O solution extracted from stained cells was detected using a microplate reader. The relative absorbance decreased significantly as the concentration of EG increased, indicating that EG suppressed adipocyte formation in a concentration-dependent manner (Figure 7B). To analyze the effect of EG on differentiation, we treated DMI with 50 μM EG under four different conditions (1; 0–6 days, 2; 0–2 days, 3; 2–4 days, 4; 4–6 days) (Figure 7C). The 3T3-L1 cells were successfully differentiated by DMI-induction in the control groups under all conditions, while EG considerably suppressed adipogenesis of 3T3-L1 cells, except for one condition (4–6 days) (Figure 7D,E). Interestingly, EG treatments for 0–2 days and 0–6 days showed similar suppressive effects on adipogenesis, whereas treatments for 2–4 days or 4–6 days showed weak or no suppressive effects. To further verify that the anti-adipogenic effects of EG were affected by PTPN6 inhibition, early adipogenic factors such as PPARγ and C/EBPα, which were decreased by PTPN6 knockdown in 3T3-L1 cells, were evaluated by Western blotting. Cells were lysed and analyzed two days after the initiation of differentiation with simultaneous EG treatment. The protein expression levels of PPARγ, C/EBPα P42, and C/EBPα P30 were significantly decreased by EG treatment in a concentration-dependent manner, showing a tendency similar to that of PTPN6 knockdown (Figure 7F,G). These results indicated that EG suppressed the early stage of adipogenesis in 3T3-L1 cells by decreasing PPARγ and C/EBPα expression levels, which could be additionally affected by the PPARγ antagonistic effect.

## 3. Discussion

The term "diabesity" was coined to suggest the deep physiological interrelation between diabetes and obesity. Management of diabetes using anti-diabetic drugs can affect body weight and several metabolic parameters, which can, in turn, have a positive or negative influence on diabetes. Therefore, the appropriate use of anti-diabesity drugs is essential for the long-term treatment of diabetes [6]. Some drugs, such as GLP-1 receptor agonists and SGLT2 inhibitors, have been proposed for the treatment of diabesity; however, side effects have been reported, and the discovery of drugs with a high safety margin and low toxicity continues to be an option of major interest [7].

In the previous studies, several phosphatases were identified as targets against diabetes or obesity, but they have not yet been reported as targets for both [21,22,43]. In this study, we identified PTPN6 as a potential target against both diabetes and obesity using siRNA. In 3T3-L1 cells, the activation of AMPK and suppression of adipogenesis was stimulated by the depletion of PTPN6, indicating that PTPN6 could be a therapeutic target for diabesity. However, the effect of PTPN6 knockdown on adipogenesis in 3T3-L1 preadipocytes was weak. Therefore, we focused on targeting PTPN6 while antagonizing PPARγ, which has also been proposed as a target for diabesity.

To identify selective PTPN6 inhibitors, an in-house library consisting of 1033 natural products was screened against 20 PTPs, which included PTPN6. As a result, 31 compounds were identified as inhibitors of PTPN6; EG was the most selective inhibitor at 20 μM concentration. Interestingly, gallate, which is structurally similar to EG, did not inhibit PTPN6. The fact that ethyl gallate best fits into the catalytic site, confirmed by docking analysis, supports the comparison of the inhibitory activities of gallate and gallate derivatives. Despite these results, as previously reported, gallate has been studied for anti-diabetic and anti-obesity properties [44,45]. To compare the effects of gallate and ethyl gallate, Oil red O staining was performed after the differentiation of 3T3-L1 cells with each compound. Ethyl gallate was more effective than gallate in suppressing adipogenesis in 3T3-L1 cells (Figure S6). We assume that these results were due to the differences in the treatment concentrations compared to that of previous studies.

The inhibition type and *K*_i_ of ethyl gallate against PTPN6 were also validated. Dixon and Lineweaver-Burk plots showed that EG is a mixed inhibitor with a *K*_i_ of 3.4 μM. Multiple docking analyses of PTPN6 were conducted to investigate the molecular basis considering allosteric inhibition of this mixed inhibition. Recently, an allosteric inhibitor of PTPN11 has been reported and used in clinical trials for genetic disorders and cancer treatment. These inhibitors bind to a narrow tunnel-like pocket composed of N-SH2, C-SH2, and the PTP domain of inactivated PTPN11 [46]. Using a similar approach, the corresponding location and inter-domain regions of PTPN6 were analyzed. However, EG and gallate derivatives did not show a promising docking pose or correlation with inhibitory activities owing to the lack of a narrow pocket in PTPN6 [39,46]. Taking these findings together, we suspect that the mixed kinetic profile of EG is derived from its combined interactions with the WPD and P loops. Additionally, docking analysis and cellular assays with PPARγ revealed that the 1,2,3-trihydroxybenzene group is a potential orthosteric binder, similar to TZD, with antagonistic activity, which could consist of a promising scaffold. EG showed PPARγ antagonistic effects with 5.4 μM IC_50_, the highest inhibition among the natural products screened for PTPN6 activity.

As EG inhibited and antagonized PTPN6 and PPARγ, respectively, the anti-diabetic effect of EG was anticipated to be induced by increasing the phosphorylation of AMPK and decreasing the phosphorylation of PPARγ at Ser273 residue. Although the regulation of 3T3-L1 glucose uptake ability by EG was evaluated by the 2-NBDG uptake assay with activation of AMPK, analysis of the phosphorylation of PPARγ at Ser273 was not performed in this study. Further studies are required to investigate whether the PPARγ antagonistic effect contributed to the glucose uptake ability of EG.

Obesity is characterized by an enlargement of adipose tissue mass, which is dependent on adipocyte hypertrophy or hyperplasia (adipogenesis) [47]. While hypertrophy means increases in size of adipocytes as a result of lipid accumulation, adipogenesis means increases in the number of adipocytes mainly in childhood or adolescence [48]. Approximately 10% of adipocyte cells are regenerated annually at all adult ages and levels of body mass index [49]. For these reasons, regulation of adipogenesis may be one of the critical pathways for controlling or reversing obesity [50].

The effects of EG on 3T3-L1 cell adipogenesis were analyzed with the help of Oil red O staining. As anticipated, EG showed a concentration-dependent anti-adipogenic effect by decreasing the protein expression levels of early adipogenic markers, such as PPARγ and C/EBPα. It might also have been affected by the inhibition of PTPN6 and PPARγ antagonistic effects, indicating that selection of the hit compound with DiFMUP-based screening and luciferase-based transactivation assay was successful. Although EG showed an anti-adipogenic effect, other important factors for obesity in terms of hypertrophy, such as lipogenesis and lipolysis [51,52], were not evaluated in this study. Assessments on those factors will increase the possibility of in vivo (or clinical) studies.

In this study, we validated the anti-diabetic and anti-obesity effects of EG in 3T3-L1 fat cells, which are widely used for in vitro studies of insulin tolerance pathways and obesity [53,54,55]. To further evaluate the anti-diabetic effects of EG, we treated C2C12 muscle cells with EG. Interestingly, EG also upregulated the glucose uptake ability of C2C12 cells, in which depletion of PTPN6 did not affect the phosphorylation of AMPK (Figures S7 and S8). Therefore, other signaling pathways must be studied further to elucidate the effects of EG on glucose uptake. In vivo assays are required to comprehensively clarify the systemic effects on diabesity.

In summary, EG was identified as a dual-targeting compound against PTPN6 and PPARγ to treat diabesity, and its efficacy was verified in 3T3-L1 cells. EG treatment stimulates glucose uptake by activating AMPK and suppressing adipogenesis in 3T3-L1 cells. The current study indicates that EG, targeting both PTPN6 and PPARγ, is a potent and novel compound against diabesity.

## 4. Materials and Methods

### 4.1. Overexpression and Purification of PTPN6

Human PTP genes were amplified by PCR using appropriate primers and the corresponding cDNAs as templates. The PCR products were digested and inserted into slightly modified pET-28a(+) or pET-30a(+) (Merck Millipore, Darmstadt, Germany), or pGEX-4T-1 vector (Cytiva, Marlborough, MA, USA), which was digested with the help of corresponding restriction enzymes. The sequence of the inserted PTP genes was identified by sequencing (BIONICS, Seoul, Korea). Each recombinant PTP construct was transformed into *Escherichia coli* Rosetta (DE3) cells (Merck Millipore, Darmstadt, Germany). Expression of recombinant human PPs was induced using isopropyl-1-thio-β-D-galactoside (IPTG). Cells were harvested by centrifugation (3570 g, at 4 °C for 10 min), washed with buffer A (50 mM Tris pH 7.5, 500 mM NaCl, 5% glycerol, 0.025% β-mercaptoethanol, and 1 mM phenylmethylsulfonyl fluoride (PMSF)), and then lysed by ultrasonication. After centrifugation (29,820 g at 4 °C for 30 min), the supernatant was incubated with an appropriate resin: cobalt affinity resin (TALON^®^; Clontech, Palo Alto, CA, USA), amylose resin (New England Biolabs, Beverly, MA, USA), or Glutathione Sepharose^™^ 4 Fast Flow (Cytiva, Marlborough, MA, USA). The protein was then eluted with buffer A containing appropriate eluent, concentrated, and stored at −70 °C. Appropriate methods for each PTP are listed in Table S2.

### 4.2. Enzymatic Assays

The enzymatic activity of each PTP was evaluated using 6,8-difluoro-4-methylumbelliferyl phosphate (DiFMUP) as the surrogate substrate. To reach within the range of the initial rate (*v*_0_) during the measurement for 10 min, the pH and concentration of each PTP were optimized and are listed in Table S3. To determine the *K*_m_ values, each PTP with optimized concentration was added to the reaction buffer (20 mM Bis-Tris at pH 6.0 or Tris at pH 7.0 and 8.0, 150 mM NaCl, 2.5 mM dithiothreitol (DTT), 0.01% Triton X-100) containing various concentrations of DiFMUP to obtain a final volume of 100 μL in a 96-well plate. Fluorescence intensities were measured once every minute for a complete 10 min duration using a Victor™ X4 Multilabel Plate Reader (Perkin Elmer, Waltham, MA, USA) at excitation/emission = 355 nm/460 nm. The initial rates of the reaction were determined using a previously described procedure for calculating the amount of product formation [56]. *K*_m_ values for each PTP analyzed based on Michaelis–Menten plots and Lineweaver-Burk plots are also listed in Table S3.

To screen a library of 1033 natural products (Chengdu Biopurify Phytochemicals Ltd., Chengdu, China) against 20 PTPs, each PTP with an optimized concentration was added to the reaction buffer with an optimized pH containing DiFMUP (2 × *K*_m_ for each PTP) after adding each compound (20 μM). For calculating IC_50_ and Hill coefficient (n_H_) values of EG on PTPN6, various concentrations of EG (80, 40, 20, 10, 5, 2.5, and 1.3 μM) were added to the reaction buffer containing DiFMUP (2 × *K*_m_; 173 μM), and then, 2 nM PTPN6 was added. The IC_50_ values were estimated using a sigmoid plot in GraphPad Prism 5 software (GraphPad Software Inc., San Diego, CA, USA). The Hill coefficient (n_H_), the degree of cooperativity, was calculated according to a previously published method [21]. To determine the inhibition constant (*K*_i_) and identify the inhibition mode of EG, various concentrations of EG (2.5, 5, 10, and 20 μM) were assayed with various concentrations of DiFMUP (260, 130, 65, and 32.5 μM). Kinetic parameters were obtained using Dixon plots for *K*_i_ and Lineweaver-Burk plots for the inhibition mode.

### 4.3. PPARγ Transactivation Assay

Briefly, CHO cells (KCLB, Seoul, Korea) were plated in 96-well plates containing Dulbecco’s modified Eagle’s medium (DMEM) (HyClone, Logan, UT, USA) supplemented with 10% charcoal-stripped FBS and 1% penicillin at a density of 1.5 × 10^4^ cells/well. After incubation for 24 h, the cells were co-transfected with pPPRE-TK-luc (Addgene #1015, Watertown, MA, USA) and pCMV6-hPPARG-GFP (Origene (Rockville, MD, USA), RG201538) using Lipofectamine 3000 (Invitrogen, Carlsbad, CA, USA) according to the manufacturer’s instructions. After 6 h of transfection, cells were treated and incubated with 20 μM compounds for screening or indicated concentrations of GW9662 or EG in the presence of 2 μM rosiglitazone for measuring IC_50_ values for another 24 h. Control cells were treated with 0.2% DMSO. Cell viability was measured using CellTiter Blue (Promega Corporation, Madison, WI, USA) for internal normalization. Firefly luciferase activity was measured using the Bright-Glo Assay System (Promega Corporation, Madison, WI, USA), according to the manufacturer’s instructions.

### 4.4. Docking Study of EG and Gallate Derivatives

All ligand docking was performed using Schrödinger Maestro 2020-4 (installed on Windows 10; New York, NY, USA). The ligands were sketched using a 2D sketcher module and prepared using the Ligprep module. We generated all possible ionized states of the ligands at physiological pH (including the neutral state) and used them for docking. The X-ray crystal structures of human PTPN6 (SHP1, PDB codes: 3PS5 and 2B3O) [38,39] dand PPARγ (PDB code: 2PRG) [40] were downloaded from the RCSB Protein Data Bank (https://www.rcsb.org/ accessed on 25 January 2022). The downloaded receptors were prepared using a protein preparation module. Missing side chains and loops were filled using Prime, and all water and unnecessary molecules were removed. The mutated Ser453 residue in PDB 3PS5 was replaced with Cys453 before minimization. The center of the grid boxes was defined by specifying the catalytic cysteine residue (Cys453, PDB code: 3PS5) and allosteric candidate region (Glu247, PDB code: 2B3O) in the crystal structure of PTPN6 and the co-crystallized ligand in PPARγ. All of the grid boxes were set to 36 Å. For docking, XP-glide in the ligand docking module was used with the default settings without constraints. Twenty poses per ligand were exported for comparison.

### 4.5. Cell Culture and Differentiation

3T3-L1 preadipocytes (Zen-Bio, Inc., Research Triangle Park, NC, USA) were maintained in high-glucose DMEM (Welgene Inc., Gyeongsan-si, South Korea), supplemented with 100 units/mL penicillin, 100 µg/mL streptomycin (Welgene Inc., Gyeongsan-si, South Korea), and 10% bovine calf serum (BCS; Thermo Fisher Scientific Korea Ltd., Seoul, Korea). Cells were passaged when they were 80–85% confluent and maintained in a sub-confluent state until they were used for differentiation. During culture and differentiation, cells were incubated at 37 °C and 5% CO_2_ in a humidified incubator (Esco Lifesciences Group Ltd., Changi, Singapore).

For the subsequent experiment, 3T3-L1 preadipocytes were cultured for 2 days in complete culture media, where a 100% confluency was reached after seeding on well plates. The cells were then incubated in DMEM supplemented with 10% FBS, 100 units/mL penicillin, 100 µg/mL streptomycin, 0.5 mM isobutylmethylxanthine (IBMX; Merck KGaA, Darmstadt, Germany), 1 μM dexamethasone (Sigma-Aldrich, Saint Louis, MO, USA), and 5 μg/mL insulin (Merck KGaA, Darmstadt, Germany). After 2 days, the medium was replaced with DMEM supplemented with 10% FBS, 100 units/mL penicillin, 100 µg/mL streptomycin, and 5 μg/mL insulin for a further 2 days, followed by culture in DMEM containing 10% FBS, 100 units/mL penicillin, and 100 µg/mL streptomycin for an additional 2–4 days.

### 4.6. RNA Interference

The method used for RNA interference has been described previously [21]. PTPN6 knockdown in 3T3-L1 preadipocytes was conducted using small interfering RNAs (siRNAs; Integrated DNA Technologies Inc., San Diego, CA, USA). 3T3-L1 preadipocytes were seeded in 6-well plates at 1.0 × 10^5^ cells/well and cultured for 24 h. After reaching 30% confluence, a final concentration of 2 nM PTPN6 siRNA duplex or negative control siRNA duplex was transfected into cells using Dharmafect (Dharmacon, GE Healthcare Korea, Songdo, Korea) according to the manufacturer’s instructions. After 48 h, a quantitative real-time-polymerase chain reaction (qRT-PCR) assay was performed to measure the efficiency of PTPN6 knockdown. The sequences of the PTPN6 siRNA duplexes are shown in Table S4.

### 4.7. Quantitative Real Time-Polymerase Chain Reaction (qRT-PCR)

Total RNA was isolated using the NucleoSpin RNA Plus Mini Kit (Macherey-Nagel, Düren, Germany), followed by treatment with DNase (Macherey-Nagel, Düren, Germany) to remove genomic DNA. Total RNA (1 µg of the total RNA) was reverse-transcribed using the High-Capacity Reverse Transcription kit (Applied Biosystems, Inc., Foster City, CA, USA), and qRT-PCR was conducted on a CFX Connect Real-Time PCR Detection System (Bio-Rad Laboratories, Inc., Hercules, CA, USA) using SsoAdvanced Universal SYBR green supermix (Bio-Rad Laboratories, Inc., Hercules, CA, USA) according to the manufacturer’s instructions. The sequences of the primers used are shown in Table S5.

### 4.8. Glucose Uptake Assay

The glucose uptake assay was performed using a previously published method, with slight modifications [57]. Fully differentiated 3T3-L1 adipocytes in µ-Slide 8 wells (Ibidi, München, Germany) were incubated overnight in low-glucose DMEM (Gibco BRL, Middlesex, UK). The cells were then treated with ethyl gallate in glucose-depleted DMEM (Gibco BRL, Middlesex, UK) for 6 h. Insulin treated independently for 30 min was used as the positive control. The fluorescent glucose analog, 2-[N-(7-nitrobenz-2-oxa-1,3-diazol-4-yl)amino]-2-deoxyglucose (2-NBDG; 100 μM), was added, and the cells were incubated for 1 h. Cells were then quickly washed with pre-cooled DPBS, and the fluorescence signal in cells was detected using confocal microscopy (LSM700 laser scanning confocal microscope, Carl Zeiss, Oberkochen, Germany). For quantitative analysis, 3T3-L1 preadipocytes were differentiated in 96-well plates independently and handled as described above for confocal microscopy, but the fluorescence signal in the cells was measured using a microplate reader (Victor^TM^ X4, PerkinElmer, Inc., Waltham, MA, USA).

### 4.9. Western Blotting

After treatment, proteins were washed twice with PBS and extracted using RIPA buffer (Sigma-Aldrich Corporation, Saint Louis, MO, USA) containing a protease inhibitor cocktail and phosphatase inhibitor cocktail (Sigma-Aldrich Corporation, Saint Louis, MO, USA). Cell lysates were centrifuged at 15,814 g, 4 °C for 20 min, and the supernatants were obtained and mixed with 5X sample buffer (GenScript, Piscataway, NJ, USA). The samples were heated for 5 min at 95 °C. After electrophoresis on a 10% sodium dodecyl sulfate-polyacrylamide gel, the proteins were transferred to a polyvinylidene fluoride (PVDF) membrane (Merck KGaA, Darmstadt, Germany) using a wet transfer system. Non-specific binding was blocked with a blocking solution containing 5% skim milk or 5% BSA and incubated overnight at 4 °C with primary antibodies: anti-SHP-1, anti-PPAR-gamma, anti-C/EBP-alpha, anti-total AMPK-alpha, anti-phosphorylated AMPK-alpha (T172), anti-total Akt, anti-phosphorylated Akt (S473) (Cell Signaling Technology, Inc., Beverly, MA, USA), and anti-β-actin (AbFrontier, Seoul, Korea). Membranes were then incubated with anti-rabbit IgG-horseradish peroxidase-conjugated secondary antibodies (AbFrontier, Seoul, Korea). Immunoreactive bands were visualized using an EzWestLumi Plus detection kit (ATTO Corporation, Tokyo, Japan), and chemiluminescence was detected using a LuminoGraph II imaging system (ATTO Corporation, Tokyo, Japan).

### 4.10. Statistical Analyses

Statistical significance (*p* < 0.05) was determined using a two-tailed unpaired *t*-test for two-group comparisons and one-way ANOVA for multiple comparisons, followed by Tukey–Kramer statistical tests (GraphPad Software, San Diego, CA, USA).

## Data Availability

All data have been provided in the manuscript. Detailed methods and additional data are available from the corresponding author upon request.

## Supplementary Materials

The following supporting information can be downloaded at: https://www.mdpi.com/article/10.3390/ijms23095020/s1.
