# Supplementary material for: Ethyl Gallate Dual-Targeting PTPN6 and PPARγ Shows Anti-Diabetic and Anti-Obese Effects
Source: Int J Mol Sci. 2022 Apr 30;23(9):5020. doi: 10.3390/ijms23095020 (PMC9105384; doi:10.3390/ijms23095020)
Supplement: Supplementary file 1 [file ijms-23-05020-s001.zip › ijms-1699784-supplementary.pdf]

Supplementary Information

Ethyl gallate dual-targeting PTPN6 and PPARγ shows anti-diabetic and anti-obese effects

Dohee Ahn [1,†], Jinsoo Kim [1,†], Gibeom Nam [1], Xiaodi Zhao [1], Jihee Kwon [2], Ji Young Hwang [1], Jae Kwan Kim [1], Sun-Young Yoon [3], and Sang J. Chung [1,2,*]

[1] School of Pharmacy, Sungkyunkwan University, Suwon 16419, Republic of Korea; ehgml94@naver.com (D.A.); neto543@naver.com (J.K.); skarlqja12@g.skku.edu (G.N.); zhaoxiaodi1019@gmail.com (X.Z.); smailemaster@naver.com (J.Y.H.); jk941002@naver.com (J.K.K.)
[2] Department of Biopharmaceutical Convergence, Sungkyunkwan University, Suwon 16419, Republic of Korea; yg1549@naver.com (J.K.)
[3] Department of Cosmetic Science, Kwangju Women's University, Gwangju 62396, Republic of Korea; dalae1104@gmail.com (S.-Y.Y.)
* Corresponding author: sjchung@skku.edu
† These authors contributed equally to this work.

List of Contents

Figure S1. The sequence of purified PTPN6. 2
Figure S2. SDS-PAGE analysis of PTPN6 purification. 3
Figure S3. Michaelis–Menten plots and Lineweaver–Burk plots. 4
Figure S4. Inhibitory activity of ethyl gallate (EG) against PTPN6. 5
Figure S5. Viability of PTPN6 inhibitors in CHO cells. 6
Figure S6. Comparison of anti-adipogenesis effects between ethyl gallate (EG) and gallic acid (GA). 7
Figure S7. Ethyl gallate (EG) treatment upregulates glucose uptake in C2C12 cells. 8
Figure S8. Knockdown of PTPN6 in C2C12 cells. 9
Table S1. List of compound names of PTPN6 inhibitors corresponding to compound numbers 10
Table S2. Details of the recombinant constructs and optimized purification methods 11
Table S3. Kinetic properties of 20 PTPs 12
Table S4. The sequence of PTPN6 siRNA 13
Table S5. The sequence of primers 14

`

MHHHHHHSSGLVPRGSGMKETAAAKFERQHMDSDEVDGDLGTMADIGSEFMVRWFHRDLSGLDAETLLKGRG
VHGSFLARPSRKNQGDFSLSVRVGDQVTHIRIQNSGDFYDLYGGEKFATLTELVEYYTQQQGVLQDRDGTIIHLK
YPLNCSDPTSERWYHGHMSGGQAETLLQAKGEPWTFLVRESLSQPGDFVLSVLSDQPKAGPGSPLRVTHIKVM
CEGGRYTVGGLETFDSLTDLVEHFKKTGIEEASGAFVYLRQPYYATRVNAADIENRVLELNKKQESEDTAKAGFW
EEFESLQKQEVKNLHQRLEGQRPENKGKNRYKNILPFDHSRVILQGRDSNIPGSDYINANYIKNQLLGPDENAKT
YIASQGCLEATVNDFWQMAWQENSRVIVMTTREVEKGRNKCVPYWPEVGMQRAYGPYSVTNCGEHDTTEYKL
RTLQVSPLDNGDLIREIWHYQYLSWPDHGVPSEPGGVLSFLDQINQRQESLPHAGPIIVHCSAGIGRTGTIIVIDML
MENISTKGLDCDIDIQKTIQMVRAQRSGMVQTEAQYKFIYVAIAQFIETTKKKLEVLQSQKGQESEYGNITYPPAM
KNAHAKASRTSSKHKEDVYENLHTKNKREEKVKKQRSADKEKSKGSLKRK

Figure S1. The sequence of purified PTPN6 with an N-terminal hexahistidine tag. The complete sequence of human PTPN6 (1-595) used for recombinant DNA construction is highlighted in red.

`

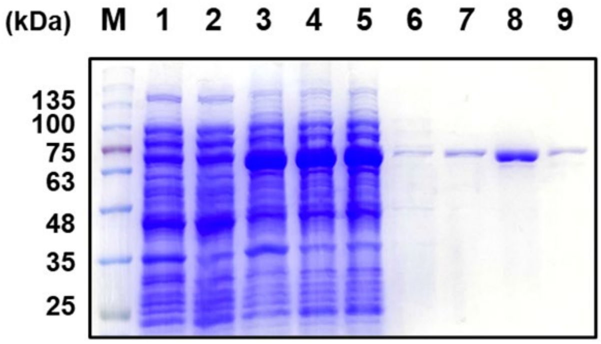

Figure S2. SDS-PAGE analysis of PTPN6 (molecular weight: 73.0 kDa) purification. PTPN6 with an N-terminal hexahistidine tag was purified using TALON affinity resin after expression in the *Escherichia coli* system. M: molecular weight marker; 1: total cell lysate before induction; 2: supernatant before induction; 3: total cell lysate after induction; 4: supernatant after induction; 5: sample after passing the supernatant through the resin; 6: sample after washing with lysis buffer; 7: sample after washing with lysis buffer containing 10 mM imidazole, 8–9: protein eluted with lysis buffer containing 100 mM imidazole.

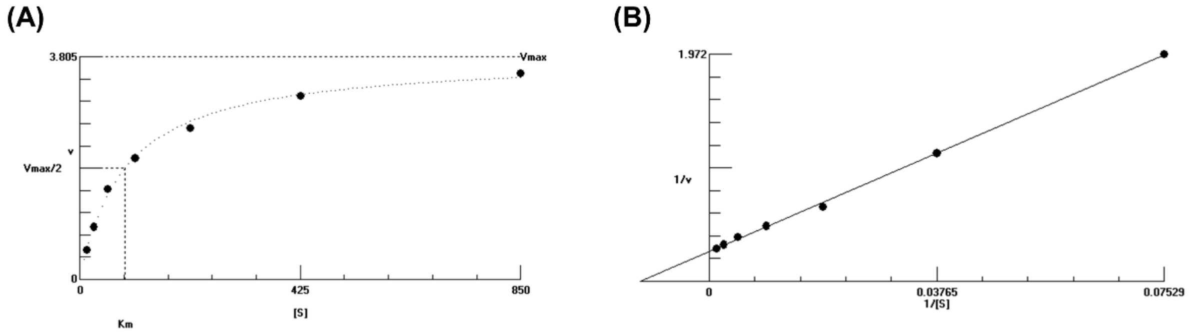

Figure S3. Michaelis–Menten plots (A) and Lineweaver–Burk plots (B) for the calculation of K_m and V_{max}. PTPN6 (2.0 nM) was added to the reaction buffer at various concentrations (850, 425, 212.5, 106.3, 53.1, 26.6, and 13.3 μM).

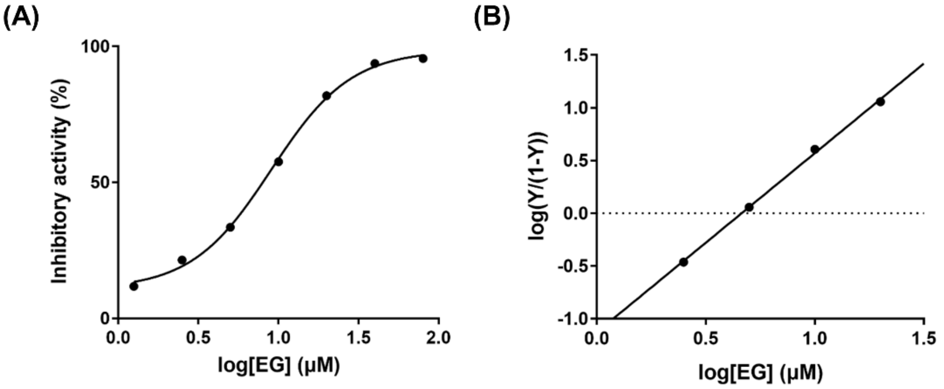

Figure S4. Inhibitory activity of ethyl gallate (EG) against PTPN6. PTPN6 (2.0 nM) was simultaneously added to the reaction buffer containing DiFMUP (173 μM) after the addition of various concentrations of ethyl gallate (80, 40, 20, 10, 5, 2.5, and 1.3 μM). (A) Half maximal inhibitory concentration (IC_{50}) value was determined to be 9.0 μM from the dose-response curves. (B) The Hill coefficient (n_H) was determined to be 1.7 from the Hill plot.

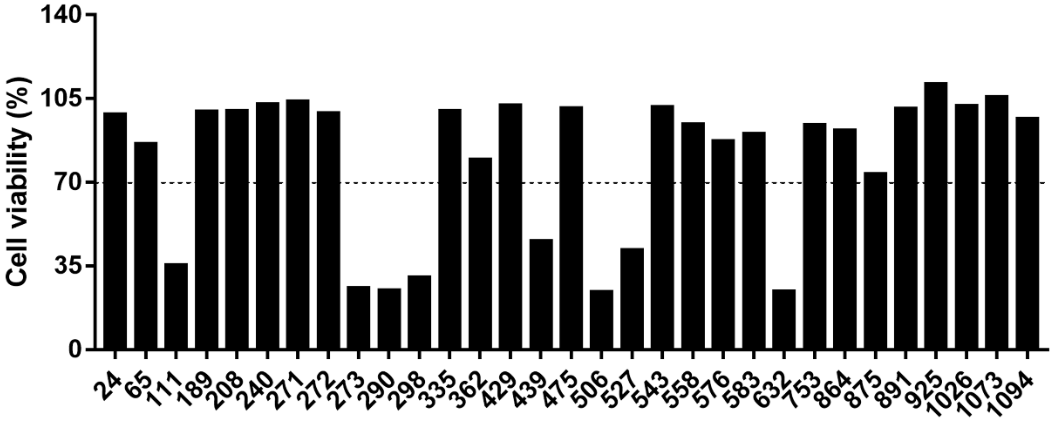

Figure S5. Viability of PTPN6 inhibitors in CHO cells. After a 6 h co-transfection with pCMV6-hPPARG-GFP and pPPRE-TK-luc, CHO cells were treated with compounds at 20 μM in the presence of 2 μM rosiglitazone and incubated for 24 h. The compound names corresponding to compound numbers are listed in Table S2.

`

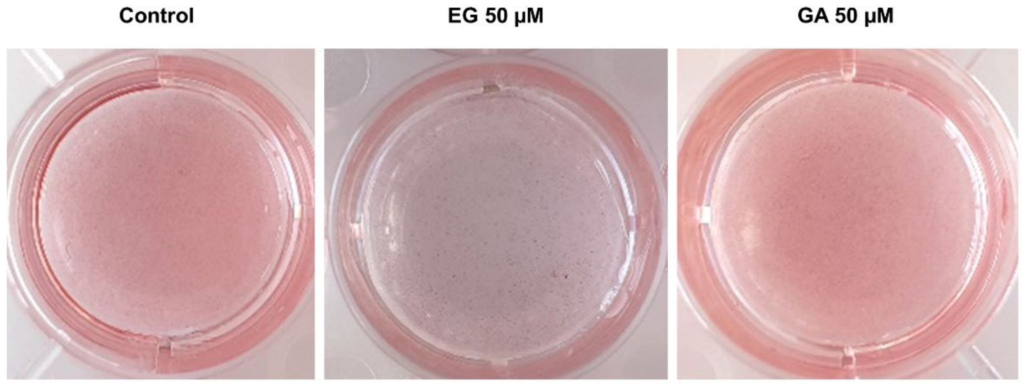

Figure S6. Comparison of anti-adipogenesis effects between ethyl gallate (EG) and gallic acid (GA). After treatment with 50 μM of each compound for 6 days during differentiation, the cells were stained with an Oil red O solution.

`

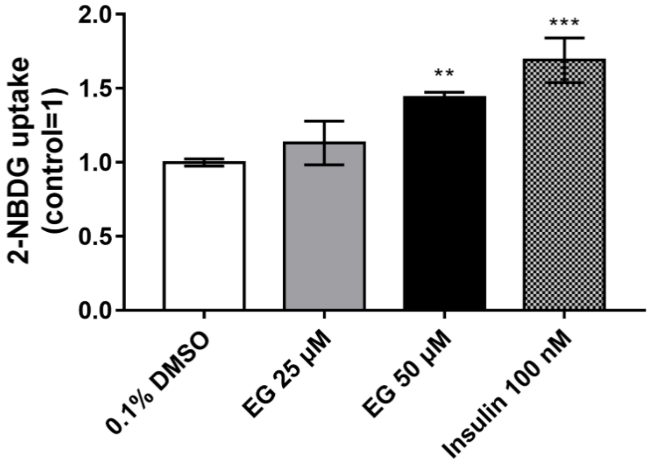

Figure S7. Ethyl gallate (EG) treatment upregulates glucose uptake in C2C12 cells. Differentiation of C2C12 cells was induced by changing the culture medium to DMEM containing 2% horse serum after reaching 90% confluency. After five days, differentiated C2C12 cells were incubated overnight with low-glucose DMEM for starvation, followed by treatment with EG (6 h) or insulin (30 min) in glucose-depleted DMEM with 0.1% DMSO. The cells were treated with 100 μM 2-NBDG for 30 min. The fluorescence intensity was detected at Ex/Em=485/535 nm using a microplate reader after washing with cold DPBS.

`

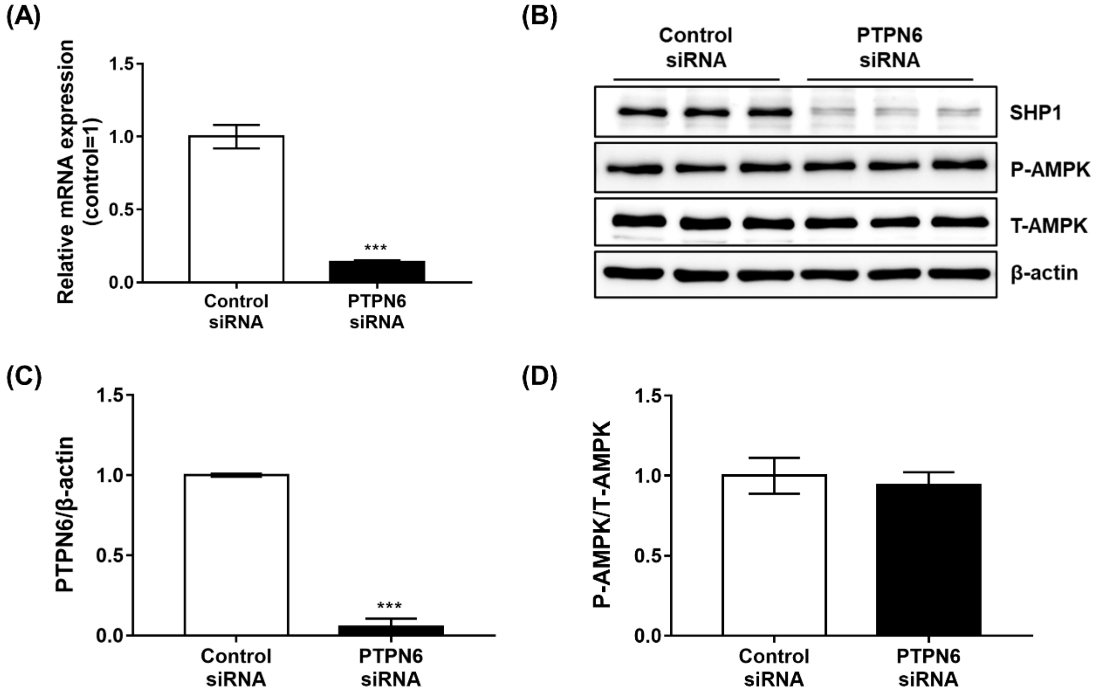

Figure S8. Knockdown of PTPN6 in C2C12 cells. C2C12 cells were transfected with PTPN6 siRNA for 48 h. After cell lysis, the expression of PTPN6 was analyzed by quantitative real-time polymerase chain reaction (qRT-PCR) (A) and western blotting (B, C). Changes in AMPK phosphorylation following the knockdown of PTPN6 were analyzed by western blotting (B). Quantification was performed and normalized to the control using CSAnalyzer 4 (C, D). Data are shown as the mean ± standard deviation (n=3). *** $p < 0.001$.

`

Table S1. List of compound names of PTPN6 inhibitors corresponding to compound numbers

Compound number	Compound name	CAS number
24	3'-hydroxygenkwanin	20243-59-8
65	Alpha-Boswellic acid	471-66-9
111	Beta,beta-Dimethylacrylshikonin	24502-79-2
189	Cyanidin chloride	528-58-5
208	Demethylzeylasteral	107316-88-1
240	Epicatechin gallate	1257-08-5
271	Gallic Acid Ethyl Ester	831-61-8
272	Gallocatechin gallate	4233-96-9
273	Gambogic acid	2752-65-0
290	Ginkgolic Acid C17:1	111047-30-4
298	Gracillin	19083-00-2
335	Icariside I	56725-99-6
362	Juglone	481-39-0
429	Myricetrin	17912-87-7
439	Neogambogic acid	93772-31-7
475	Parishin A	62499-28-9
506	Polyphyllin II	50773-42-7
527	Punicalin	65995-64-4
543	Ricinoleic acid	141-22-0
558	Salvianolic acid C	115841-09-3
576	Scutellarein	529-53-3
583	Sennoside B	128-57-4
632	Timosaponin AIII	41059-79-4
753	3-Acetyl-11-keto-beta-boswellic Acid	67416-61-9
864	Dehydroandrographolide Succinate Potassium Salt	76958-99-1
875	Erucic acid	112-86-7
891	Agaricic acid	666-99-9
925	Methyl syringate	884-35-5
1026	Hederagenin	465-99-6
1073	Quercetin Dihydrate	6151-25-3
1094	Tanshinone IIA sodium sulfonate	69659-80-9

`

Table S2. Details of the recombinant constructs and optimized purification methods

Gene name	Vector	Tag	IPTG concentration	Induction temperature & time	Resin	sequence
PTPRJ	pET 30a DEVD	N-terminal His	0.1 mM	18 °C, 16 h	TALON®	1019-1311 a.a
PTPN1	pET 28a DEVD	C-terminal His	0.1 mM	18 °C, 16 h	TALON®	1-299 a.a
PTPN2	pET 28a DEVD	N-terminal MBP and C-terminal His	0.1 mM	18 °C, 16 h	TALON®	1-381 a.a
PTPN3	pET 28a DEVD	N-terminal MBP and C-terminal His	0.1 mM	18 °C, 16 h	TALON®	403-913 a.a
PTPN6	pET 30a DEVD	N-terminal His	0.1 mM	18 °C, 16 h	TALON®	Full
PTPN7	pET 28a DEVD	N-terminal MBP and C-terminal His	0.1 mM	18 °C, 16 h	TALON®	281-533 a.a
PTPN9	pET 28a DEVD	N-terminal His	0.1 mM	18 °C, 16 h	TALON®	277-581 a.a
PTPN11	pET 30a DEVD	N-terminal His	0.1 mM	18 °C, 16 h	TALON®	1-526 a.a
PTPN12	pET 28a DEVD	N-terminal MBP and C-terminal His	0.1 mM	18 °C, 16 h	TALON®	1-334 a.a
PTPN14	pET 28a DEVD	N-terminal MBP and C-terminal His	0.1 mM	18 °C, 16 h	TALON®	908-1187 a.a
PTPN18	pET 28a DEVD	N-terminal MBP and C-terminal His	0.1 mM	18 °C, 16 h	TALON®	1-300 a.a
PTPN21	pET 28a DEVD	N-terminal MBP and C-terminal His	0.1 mM	18 °C, 16 h	TALON®	839-1174 a.a
DUSP9	pET 28a DEVD	N-terminal MBP and C-terminal His	0.1 mM	18 °C, 16 h	TALON®	Full
DUSP3	pET 28a DEVD	N-terminal MBP and C-terminal His	0.1 mM	18 °C, 16 h	TALON®	1-181 a.a
DUSP12	pET 28a DEVD	N-terminal MBP and C-terminal His	0.1 mM	18 °C, 16 h	TALON®	1-340 a.a
DUSP14	pET 28a DEVD	N-terminal MBP and C-terminal His	0.1 mM	18 °C, 16 h	TALON®	1-198 a.a
DUSP15	pET 28a DEVD	N-terminal MBP and C-terminal His	0.1 mM	18 °C, 16 h	TALON®	1-235 a.a
DUSP18	pET 28a DEVD	N-terminal MBP and C-terminal His	0.1 mM	18 °C, 16 h	TALON®	1-188 a.a
DUSP22	pET 28a DEVD	N-terminal MBP and C-terminal His	0.1 mM	18 °C, 16 h	TALON®	1-184 a.a
SSH2	pET 28a DEVD	N-terminal MBP and C-terminal His	0.1 mM	18 °C, 16 h	TALON®	298-449 a.a

`

Table S3. Kinetic properties of 20 PTPs

PTP name	pH	Concentration (nM)	K_m (μM)
PTPRJ	6	0.03	66.4
PTPN1	6	0.15	21.6
PTPN2	6	0.25	20.0
PTPN3	6	1	2.0
PTPN6	7	2	86.3
PTPN7	6	0.5	25.5
PTPN9	7	0.15	110
PTPN11	6	10	100
PTPN12	6	0.6	75.8
PTPN14	6	30	423.7
PTPN18	6	0.5	5.5
PTPN21	8	20	46.7
DUSP9	7	200	35.0
DUSP3	6	1	100.0
DUSP12	6	0.1	19.0
DUSP14	6	15	55.0
DUSP15	7	45	9.1
DUSP18	7	37	29.0
DUSP22	6	10	13.0
SSH2	6	10	215.3

`

Table S4. The sequence of PTPN6 siRNA

	**Sequence (5'-3')**
Sense	CAGGAGGUAAAGAAUCUACACCAAC
Antisense	GUUGGUGUAGAUUCUUUACCUCCUGCU

`

Table S5. The sequence of primers

Gene	Forward (5'-3')	Reverse (5'-3')
Mouse *PTPN6*	CTTGGCAGGAGAACACTCGT	GCCATATCTCCCGAACCAGG
Mouse *GAPDH*	GGCAAATTCAACGGCACAGT	GGCGGAGATGATGACCCTTT

`
